# Multimodal Phenotyping of Alzheimer’s Disease with Longitudinal Magnetic Resonance Imaging and Cognitive Function Data

**DOI:** 10.1038/s41598-020-62263-w

**Published:** 2020-03-26

**Authors:** Yejin Kim, Xiaoqian Jiang, Luca Giancardo, Danilo Pena, Avram S. Bukhbinder, Albert Y. Amran, Paul E. Schulz, Alzheimer’s Disease Neuroimaging Initiative

**Affiliations:** 1 School of Biomedical Informatics, University of Texas Health Science Center at Houston, Houston, Texas USA; 2 Department of Neurology, the McGovern Medical School, University of Texas Health Science Center at Houston, Houston, Texas USA; 3 Department of Diagnostic and Interventional Imaging, the McGovern Medical School, University of Texas Health Science Center at Houston, Houston, Texas USA

**Keywords:** Computer science, Neurodegenerative diseases

## Abstract

Alzheimer’s disease (AD) varies a great deal cognitively regarding symptoms, test findings, the rate of progression, and neuroradiologically in terms of atrophy on magnetic resonance imaging (MRI). We hypothesized that an unbiased analysis of the progression of AD, regarding clinical and MRI features, will reveal a number of AD phenotypes. Our objective is to develop and use a computational method for multi-modal analysis of changes in cognitive scores and MRI volumes to test for there being multiple AD phenotypes. In this retrospective cohort study with a total of 857 subjects from the AD (n = 213), MCI (n = 322), and control (CN, n = 322) groups, we used structural MRI data and neuropsychological assessments to develop a novel computational phenotyping method that groups brain regions from MRI and subsets of neuropsychological assessments in a non-biased fashion. The phenotyping method was built based on coupled nonnegative matrix factorization (C-NMF). As a result, the computational phenotyping method found four phenotypes with different combination and progression of neuropsychologic and neuroradiologic features. Identifying distinct AD phenotypes here could help explain why only a subset of AD patients typically respond to any single treatment. This, in turn, will help us target treatments more specifically to certain responsive phenotypes.

## Introduction

Alzheimer’s disease (AD) is the most common form of dementia. It is a progressive neurodegenerative disorder associated with cognitive decline and atrophy seen on Magnetic Resonance Imaging (MRI) of the brain^1^. It has become a major public health concern because of its increasing prevalence, chronicity, caregiver burden, and high personal and financial costs of care^2^.

AD is clinically very heterogeneous, varying between patients in terms of cognitive symptoms, test findings, and rates of progression^3^. It also varies neuroradiologically in terms of atrophy on MRI Memory deficits, caused by pathological changes in structures of the medial temporal lobe^4,5^, are typically regarded as the earliest and most salient symptom of AD^6,7^, but this is not invariably the case^8^. Instead, patients may present with visuospatial or language disturbance, or apraxia^9^, likely reflecting regional differences in the underlying neuropathology^8,10–12^. It can also present as a single area of cognitive impairment without a change in activities of daily living, in which case this prodromal AD is referred to as mild cognitive impairment, or MCI.

Several recent treatment trials for AD have shown efficacy in a subset of patients, but not all patients. We hypothesize that there are subsets of AD patients who respond differently to treatments. Furthermore, we hypothesize that these subsets may correspond to different AD phenotypes revealed by analyzing the clinical and MRI variability in AD presentation.

Recent advances in computational phenotyping methodologies have introduced data-driven phenotyping of AD and related dementias. This computational approach is automated and non-biased, high-throughput, and can handle vast amounts of noisy healthcare data^13–15^. One method has examined patterns of cortical atrophy on brain imaging to suggest AD subtypes^16^. Others have used factor analysis and hierarchical clustering to group AD patients according to cognitive features^8^. Statistical analysis using mixed effects models and multiple linear regression is used to confirm association between cognitive visual rating scales and neuroradiologic subtypes^17^. So far, none of the previous research on phenotyping uses both neuroradiologic features from imaging and neuropsychological features from cognitive tests. This lack of multi-modal view motivated us to develop a novel multi-modal computational phenotyping model that integrates neuroimaging and cognitive features. We hypothesize that by combining these two sources of data, phenotyping outputs for neurodegeneration of AD would be more robust. We also focused on longitudinal progression of neuroimaging and clinical assessments because AD is a progressive disease and understanding the neurodegeneration is the main outcome of interest in AD research^3^. Therefore, our objective is to develop and use the computational phenotyping method for multi-modal analysis of changes in cognitive scores and MRI volumes of AD patients to test for there being multiple AD phenotypes.

**Figure 1:**
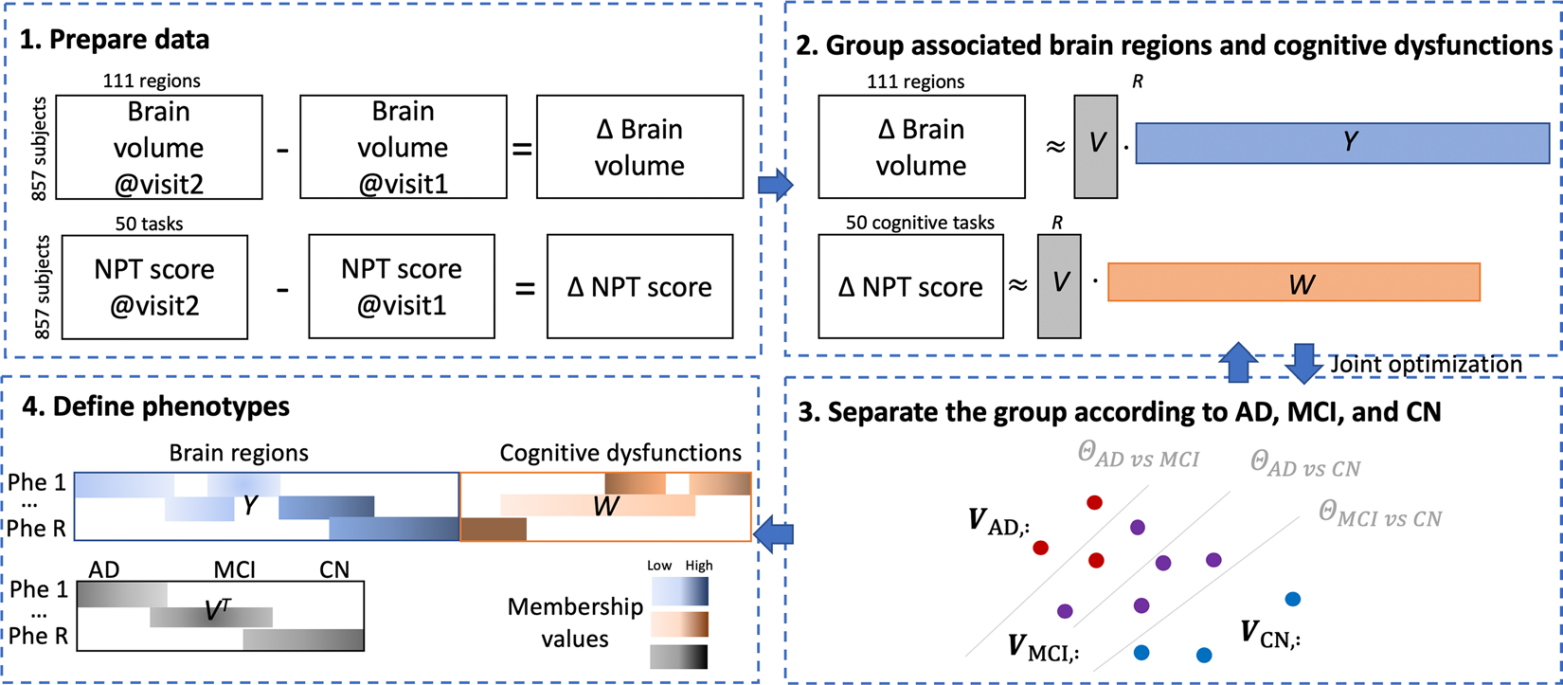
Workflow from Data Preprocessing to Interpretation of Phenotypes. NPT = Neuropsychological tests. *R* = the number of phenotypes. (1) Prepare data: We analyzed the longitudinal changes between two visits in the brain volume of each brain region and the NPT scores. Brain volume changes were M: = Δ Brain volume = Brain volume at visit 2 – Brain volume at visit 1. NPT score changes were X: = Δ NPT score = NPT score at visit 2 – NPT score at visit 1. (2) Group using matrix factorization: We derived phenotypes as a set of associated brain regions and cognitive dysfunction. Brain volume changes M is decomposed into V (subject’s membership) and Y (brain region’s membership). NPT score changes X is decomposed into V (subject’s membership) and W (cognitive task’s membership). We used coupled nonnegative matrix factorization to harmonize the two information. (3) Sep arate the groups by multi-label support vector machines: We encouraged the V (subject’s membership), Y, and W to reflect subject’s disease stages (AD, MCI, and CN). The matrix factorization and the support vector machines are jointly optimized. (4) Define phenotypes: We analyzed clinical relevance of the cognitive dysfunction and related brain volume loss.

In this study, the computational phenotyping method is based on coupled nonnegative matrix factorization of brain volume loss and deteriorated cognitive dysfunction, together with separating regularization in terms of disease stage (Fig. 1). The phenotypes are defined as a set of decreased brain regions and decreased cognitive function with different weights or membership values. Subjects also have multiple phenotypes with different membership values.

## Results

### Patient cohort

From Alzheimer’s Disease Neuroimaging Initiative (ADNI) database, we included AD, MCI and cognitively normal (CN) subjects that have eligible imaging sessions and clinical assessments (Table 1). 857 subjects in the ADNI dataset had more than one imaging session that occurred at least six months apart. AD, MCI, and CN subjects showed different longitudinal progression (Table 2) and transitions from MCI or CN to AD (Supplementary Table S1). Informed consent was obtained for all subjects, and the study was approved by the relevant institutional review board at each data acquisition site (for up-to-date information, see http://adni.loni.usc.edu/wp-content/themes/freshnews-dev-v2/documents/policy/ADNI_Acknowledgement_List%205-29-18.pdf). All methods were performed in accordance with the relevant guidelines and regulations.

**Table 1:** Description of the ADNI cohort dataset at first visit. AD = Alzheimer’s dementia, MCI = mild cognitive impairment, CN = cognitively normal controls.

Basic demographics (based on visit 1)	AD	MCI	CN
Number of Patients	213	322	322
Age, years (mean (s.d.))	74.6 (7.6)	74.5 (7.3)	74.9 (5.8)
Male gender (n (%))	108 (50.7%)	203 (63%)	164 (50.9%)
Education, yr	15.24 (2.73)	15.69 (2.89)	16.4 (2.67)
Marital status			
Married	175 (82.2%)	258 (80.1%)	214 (66.5%)
Widowed	23 (10.8%)	37 (11.5%)	54 (16.8%)
Divorced	10 (4.7%)	22 (6.8%)	32 (9.9%)
Never married	5 (2.3%)	5 (1.6%)	21 (6.5%)
Number of ApoE4 carriers			
No	63 (29.6%)	147 (45.7%)	233 (72.4%)
One APOE4 allele	105 (49.3%)	129 (40.1%)	77 (23.9%)
Two APOE 4 alleles	45 (21.1%)	42 (13.0%)	11 (3.4%)
Time between two imaging sessions (mean (s.d.)) (years)	1.6 (0.6)	3.8 (2.6)	4.8 (2.9)

**Table 2:** Longitudinal progression of ADNI cohort.

Disease stage at visit 1	AD		MCI		CN	
	Visit 1 (n = 213)	Visit 2 (n = 384)	Visit 1 (n = 322)	Visit 2 (n = 183)	Visit 1 (n = 322)	Visit 2 (n = 277)
FDG-PET (mean (s.d))	1.07 (0.14)	1.01 (0.16)	1.2 (0.12)	1.12 (0.16)	1.31 (0.11)	1.26 (0.14)
CDR-SB	4.37 (1.59)	6.57 (2.94)	1.55 (0.86)	4.26 (3.71)	0.03 (0.12)	0.5 (1.39)
RAVLT immediate	23.25 (7.18)	18.68 (8.26)	31.09 (9.22)	25.32 (12.27)	44.7 (9.72)	42.85 (12.06)
RAVLT learning	1.72 (1.77)	1.49 (1.69)	3.33 (2.36)	2.62 (2.29)	5.91 (2.3)	5.17 (2.65)
RAVLT forgetting	4.49 (1.72)	4.08 (2.06)	4.59 (2.2)	4.05 (2.29)	3.71 (2.79)	3.72 (2.81)
LDEL total	1.26 (1.79)	0.84 (2.04)	3.93 (2.74)	3.92 (4.89)	13.34 (3.26)	13.29 (4.89)
DIGIT score	27.9 (12.31)	22.81 (14.32)	37.69 (11.12)	32.46 (13.61)	45.96 (10.66)	44.86 (12.87)
Trail B score	192.36 (85.21)	220.58 (89.18)	125.94 (72.44)	165.53 (104.62)	85.04 (42.99)	95.93 (55.52)
Ventricles, $m{m}^{3}$	46499.51 (22953.63)	52370.72 (24362.76)	42051.86 (21748.35)	52551.46 (25301.08)	33541.74 (16974.45)	40761.95 (19629.52)
Hippocampus, $m{m}^{3}$	5635.56 (986.56)	5300.64 (1068.79)	6390.79 (1086.61)	5776.29 (1175.13)	7351.41 (869.71)	6914.75 (983.24)
Whole Brain, $m{m}^{3}$	965948.3 (115387.73)	936315.7 (113321.24)	996207.8 (105351.95)	956487.3 (105646.97)	1024226 (105334.28)	992703.1 (108390)
Entorhinal, $m{m}^{3}$	2786.12 (681.65)	2558.33 (679.19)	3309.26 (752.82)	2985.22 (799.2)	3800.91 (639.1)	3625.8 (684.76)
Fusiform, $m{m}^{3}\,$	15238.1 (2610.82)	14286.5 (2717.05)	16438.82 (2328.43)	15392.79 (2541.99)	17652.72 (2404.69)	17039.53 (2595.64)
Mid Temp, $m{m}^{3}$	16940.57 (3086.25)	15700.37 (3152.04)	18693.25 (2927.51)	16960.81 (3347.8)	20089.13 (2672.98)	19203.15 (2848.84)
ICV, $m{m}^{3}$	1532939 (175896.59)	1534174 (174868.9)	1572611 (163710.12)	1579756 (172889.54)	1524544 (154553.21)	1528301 (161108.2)
Amyloid beta, pg/ml	672.9 (310.61)	526.76 (182.85)	835.05 (419.06)	710.51 (409.3)	1187.06 (448.89)	1188.7 (448.57)
Tau, pg/ml	369.25 (138.03)	388.98 (161.34)	314.67 (115.32)	324.83 (133.22)	236.01 (91.05)	256.7 (102.02)
P-Tau, pg/ml	36.85 (15.23)	39.34 (18.43)	31.18 (13.3)	31.14 (15.63)	21.77 (9.37)	23.84 (10.82)

Abbreviations: FDG-PET = F-fluorodeoxyglucose positron emission tomography. CDR-SB = Clinical dementia rating–sum of boxes. RAVLT = Rey auditory verbal learning test. LDEL = Logical memory delayed. DIGIT = Digit Symbol Substitution Test. ICV = Intracranial volume. P-Tau = Phosphorylated Tau.

### Four computational phenotypes with different progression and combination

After deriving *R* = 30 phenotype candidates, we selected four representative phenotypes. We first filtered out less discriminative 21 phenotypes based on statistical significance, i.e., *p*-value of multivariate logistic regression on classifying AD and/or MCI vs CN (Supplementary Table S2) and distribution of AD, MCI, and CN subjects who have high membership values to the phenotypes (Figure S1). Note that the membership values refer to the amount or weight that the subjects, brain regions, or tasks contribute to define the phenotype. The phenotype membership values are analogous to the membership in fuzzy clustering. To confirm the discriminative performance of selected phenotypes with respect to the disease stages, we compared coefficient and *p*-value of logistic regression assuming that the phenotypes are used as predictors to predict disease stage (either AD vs MCI; MCI vs CN; or AD/MCI vs CN) at visit 1. Note that, although the time duration between two visits varied depending on the disease stage, the time duration was taken into account in the logistic regression model to cancel out the bias effect of different duration. We also examined the number of disease stage transitions between two visits using confusion matrix (Supplementary Table S3). With the remaining 9 phenotypes we reviewed their related cognitive function, brain regions, and biological variables and selected four phenotypes with distinct characteristics. The four phenotypes showed distinct cognitive decline pattern (Table 3). Each phenotype had a set of brain regions that show significant volume loss and/or cognitive function that shows increase in severity.

**Table 3:** Representative four phenotypes and one normal aging characteristic with its progression between two visits.

Phenotype definition (and prevalence)	Declined NPT tasks & volume loss on brain regions	Impaired cognitive areas	Amyloid beta (pg/ml)	Tau (pg/ml)	P-Tau (pg/ml)	CDR-SB
Phenotype 1 Memory decline (40.3%, n = 345)	Word recall; Writing a check; Paying bills, or balancing checkbook; Lh caudal anterior cingulate		816.6$\to$865.3	327.2 $\to$310.9	32.4 $\to$30.3	2.3$\to$5.5*
Phenotype 2 Language deficit (18.9%, n = 162)	Comprehension; Orientation; Word Finding; Spoken language		709.3$\to$ 837.2 *	355.1 $\to \,$314.8*	35.1 $\to \,$30.6*	4.2$\to$7*
Phenotype 28 Progressed AD (36.8%, n = 315)	Writing checks, paying bills, or balancing checkbook; Recall instructions		796.3 $\to \,$868.1*	332.4 $\to$316.7	32.9 $\to \,$30.9	3.1$\to$5.8*
Phenotype 21 Visuospatial planning dysfunction (34.9%, n = 299)	Number cancellation; Ideational Praxis; Naming		83 4.1$\,\to$862.5	32 1.3 $\to$ 312.2	31.6 $\to \,\,$30.2	2.3$\to$5.8*
Phenotype 4 Normal aging (99.9%, n = 856)	Paying attention to and understanding TV program, book, or magazine Wm rh pericalcarine; wm rh lingual; wm lh lingual; wm rh insula; wm rh parahippocampal; left hippocampus (and other 45 areas)		918.9$\to$895.4	301.7 $\to$304.3	29. 4 $\to \,$29.5	1.6$\to$3.7*

We listed the neuropsychological tests (NPT) and brain regions with highest involvement or membership on each phenotype. Prevalence is computed as the number of patients who have the characteristic of the phenotype (i.e., membership value > 10^−5^)/total number of patients. We plotted five cognitive areas (memory, visuospatial, orientation, executive, and language function) using ADAS-cog (i.e., Q1, Q4, and Q9 for memory; Q3 and Q6 for visuospatial; Q7 for orientation; Q2 for executive; Q8, Q10, Q11, Q12, and Q5 for language). We normalized the partial sum of cognitive scores by dividing it by maximum values. We presented the two visits’ mean values of various biomarkers (incl uding Amyloid-beta, Tau, P-Tau, and CDR-SB) to see underlying progression. Due to limited space, comprehensive variables including demographics, ApoE allele, and RAVLT for each phenotype can be found in Supplementary Table S3. We examined statistical significance on the change of biomarkers using weighted *t*-test, where the weights are obtained from the patient’s membership value to each phenotype.

Abbreviations: CDR-SB = Clinical dementia rating–sum of boxes; P-Tau = Phosphorylated Tau; wm = white matter; rh = right hemisphere; lh = left hemisphere; * if *p*-value <0.1 for weighted *t*-test to evaluate the values from first and second visits change significantly.

## Discussion

The objective of this study was to develop and validate a multi-modal phenotyping method to test the hypothesis that there are identifiable AD phenotypes that are based on progressive loss of brain regions and associated loss of cognitive functions.

We developed a phenotyping method using coupled nonnegative matrix factorization with a supervised support vector machines regularizer and shrinking regularizer. We compared the interpretability and discriminability of the phenotyping method with baseline models. Using this method, we derived phenotypes that consist of relevant brain regions and cognitive functions that show similar longitudinal loss.

The produced phenotypes in this study do show that subtypes of ADNI patients decline in cognitively distinguishable ways. Phenotype 1 is characterized chiefly by a decline in the ability to recall words on cognitive testing, which is a common hallmark of AD and one of the ways AD patients can be functionally differentiated from those that are cognitively normal^18^. However, there were also declines in the volume of the caudal anterior cingulate gyrus and in the ability to write checks or pay bills. The anterior cingulate gyrus has been shown to be associated with motivation, decision making, cost-benefit calculations, and conflict and error monitoring^19^. While no studies to date have correlated certain instrumental activities of daily living with specific aspects of cognition, motivation and recognition of consequences are both theoretically involved in the act of paying bills.

The areas of decline of the AD patients in Phenotype 2 most closely resemble that of logopenic progressive aphasia, a form of language-deficit dementia most commonly attributed to an underlying Alzheimer’s pathology. This particular disease subtype is marked mainly by gradual loss in the ability to repeat phrases or name objects, however single-word comprehension is normally conserved^20^. And while single-word comprehension is spared, understanding longer phrases and sentences is significantly more difficult for logopenic patients. Comprehension of longer sentences requires patients to retain more information to process, and thus is impaired because of the short-term memory loss that is common in AD. In Phenotype 2 the ADAS and MMSE criteria that decline in between the visits for these patients are all associated with naming objects, word repetition, and comprehension. Orientation deficits were also clustered in Phenotype 2, and are not unexpected given the association of AD with degeneration of the parietal lobe. This is interesting due to the proximity of the parietal lobe to Wernicke’s area in the temporal region which is often involved in language comprehension difficulties as well. This pattern of decline may suggest that the decline over the two-year period involved the posterior medial temporal lobe as well as the parietal lobe^21^. Patients in Phenotype 2 appeared to decline in cognitive areas more related to language, orientation, and comprehension. Scoring of several areas of cognition within this phenotype showed decline across the board in executive, visuospatial, language, and orientation. This is not unexpected considering the communication difficulty when trying to assess patients with language disorders. Other areas of cognition can appear to be reduced because a patient may not understand test instructions or be able to answer properly. This has been a common criticism of tests such as the MMSE in the past^22^. In fact, it was expected that patients with strong language decline over the two year period would test as having global cognitive decline.

The criteria clustered in Phenotype 28 include a mixture of Phenotypes 1 and 2 in regards to the decline in orientation and an inability to balance a checkbook. Significant global impairments in cognitive function were noted in other examinations of this group suggesting that specific changes may be masked by language dysfunction as well, matching that of Phenotype 2. Examination of molecular markers between the three groups did not display a conclusive relationship although Phenotypes 2 and 28 both saw increases in amyloid-beta in cerebrospinal fluid. The significance of this finding is unknown as amyloid-beta quantities have been historically used for diagnosis of AD rather than progression^23^. CSF phosphorylated tau and total tau have also not been shown to be associated with Alzheimer’s severity^24^.

Phenotype 4 was shared by 99.9% of patients analyzed in this study. Its inclusion of AD, MCI, and CN patients suggests that it may represent baseline age-related cognitive decline. Other studies of this same patient database have identified the clustered changes in Phenotype 4 as belonging specifically to Alzheimer’s patients^25^. Structural degeneration of the clustered areas results in damage to the hippocampal-prefrontal cortex pathway which was also found in other studies of AD patients. A little less than half were diagnosed with AD at the second visit, but all of the clustered patients are associated with these patterns. Moreover, there was no association of these patients with degeneration of the prefrontal cortex which serves as an enhancer of the encoding of memory^26^. Based on the declining structures clustered in this phenotype, it would be more accurate to say that the pathway between the prefrontal cortex and the areas which do encode working memory as well as the areas themselves were degenerating in Phenotype 4 patients. The specific pattern of degenerating structures suggests that the functional deficits observed in the cognitive testing in this cluster were due to an Alzheimer’s-like pathology, at least according to prior studies of this patient database.

While it is possible that all of the patients in Phenotype 4 are undergoing AD-like changes, it is more likely that this pattern is in fact representative of baseline age-related cognitive changes. A recent study comparing young versus older adult connectomes showed declines in the functionality of this same dorsal prefrontal-attention axis even in healthy elderly patients while executive, orientation, visuospatial, and language areas were spared^27^. The prevalence of Phenotype 4 suggests that it is a common degenerative pathway in all of this study’s patients, including the cognitively normal ones. Comparisons of the CDR with other phenotypes’ CDRs show that the average CDRs of Phenotype 4 were less than that of the other groups (i.e., weighted *t*-test’s *p*-values <0.03 with P1, P2, P28, P21 for both visit 1 and visit2). Less severe CDRs, high prevalence across both healthy and cognitively impaired patients, and the specific pattern of degeneration altogether suggests that Phenotype 4 may represent age-associated decline.

Phenotype 21 had similar types of structural and functional changes to Phenotype 4 although structural changes were weakly clustered. The primary associated features in Phenotype 21 were a decline in performance on the number cancellation test and marked decreases in ideational praxis which represents a decline in visuospatial planning, a cognitive function primarily associated with the parietal lobe^28^. Parietal atrophy is a recognized feature of a rare variant of non-amnestic Alzheimer’s disease referred to as posterior cortical atrophy (PCA) which involves dysfunction of the occipital and parietal lobes^29^. Although the relative rarity of PCA suggests that the likelihood that 34.9% of the ADNI patients had this variant is very low, they were experiencing non-amnestic visuospatial cognitive decline. Unaffected measures of memory dysfunction over the two-year period suggests that it is likely that these patients did not have amnestic AD.

A limitation of this study is that the brain’s regional volume loss was less captured in phenotype definitions compared to cognitive task scores. The time between two imaging sessions were different based on the disease stages. Cognitively normal subjects have 4.8 years apart, whereas AD subjects have 1.6 years apart due to active follow up (Table 1). Although we reduced the bias from the time difference when predicting the disease stage as incorporating the difference in the regression model, the phenotype definition itself still contained bias from the time difference. AD subject’s significant brain volume changes might not be observed due to the relatively short time difference, consequently the phenotypes were sometimes defined with only cognitive changes.

The main contribution of this study is to demonstrate that one can derive phenotypes of AD using longitudinal neuroimaging features and cognitive assessments, which are complementary sources of information that follow different distributions. In addition, this study examined changes over time, and was not based on static, initial findings. Prior studies that have developed computational phenotyping methodologies focus on one modality of data source, either on brain imaging^16^ or cognitive functioning (via NPT scores)^8^ as cross-sectional studies. To the best of our knowledge, our multi-modal and longitudinal phenotyping method is the first of its kind. This multi-modality allows us to capture phenotypes with various combinations of clinical presentations and neuroradiologic features. The longitudinal approach allows us to compare the progression of clinical presentation and neuroradiologic features. The harmonization of multi-modal and longitudinal approaches into one framework enables us to have a unique perspective on AD phenotyping research in considering both combinations and progressions. Moreover, it has been difficult to understand why only a subset of AD patients typically respond to any single treatment. Identifying distinct AD phenotypes here could help explain those results, i.e. only certain AD phenotypes may be responding to each treatment. This, in turn, will help us target treatments more specifically to certain responsive phenotypes.

## Materials and Methods

### Dataset

We used the Alzheimer’s Disease Neuroimaging Initiative (ADNI) database to build the phenotyping model. ADNI is a multisite study to define the progression of AD. It collects and validate AD’s progression data including MRI, cognitive tests, and blood biomarkers from AD, MCI, and CN subjects. There were 36.2% missing values in the clinical variables in ADNI.

### MRI acquisition/processing

Structural MRI scans provide a visual depiction of the size of white and gray matter structures at a single time-point. MRI scans at multiple time-points are therefore a useful tool to assess longitudinal volumetric changes. In this study, the first and last recorded visits for each subject were used for the analysis. The Brain Imaging Data Structure format was used to create a data structure for the longitudinal pipeline^30,31^. T1-weighted images from MRI scans were acquired from the LONI Image Data Archive on November 2018^32^. Cortical and subcortical volumetric segmentations were performed using the FreeSurfer Longitudinal Processing pipeline v. 6.0^33,34^. This pipeline uses an unbiased within-subject template space to register the images at multiple time points; this method reduces the intra-subject volume estimation error that might occur when only inter-subject templates are used, as is typically done in most cross-sectional studies. The pipeline automatically segmented the cortical and subcortical areas and normalized the voxel intensity. It improved the segmentation quality by correcting the boundaries between white/gray matter and gray matter/cerebrospinal fluid (CSF). In our analysis we included all 111 regions available in the standard FreeSurfer pipeline^35^.

### Neuropsychological assessments

Neuropsychological tests (NPTs) are a valuable source of information for cognitive dysfunction of AD. NPTs are widely used as a first step in the diagnosis of AD. These tests characterize AD by identifying the most salient and earliest cognitive and behavioral symptoms, thereby also providing information on the staging and tracking of the disease^36^. For example, the Alzheimer’s Disease Assessment Scale-Cognitive subscale (ADAS-Cog) is regularly used to assess the severity of core cognitive findings in patients with AD^37^; It consists of 13 tasks that are designed to assess various cognitive domains, including memory, language, praxis, and attention^38^. The Mini-Mental State Examination (MMSE) is the most commonly administered psychometric screening assessment of global cognitive function^39,40^. The MMSE is generally used to screen patients for cognitive impairment and to track changes in cognitive functioning over time^39,40^. The Functional Activity Questionnaire (FAQ) is also commonly used to measure impairment in instrumental activities of daily living^41^.

As stand-alone, one-time administrations of each of the aforementioned NPTs sometimes does not accurately identify AD and MCI patients^37,42–45^, neuroimaging as a complementary data source can potentially supplement the disease identification. We used 50 individual NPT subtasks (13 ADAS-cog, 27 MMSE, and 10 FAQ) as variables. Missing values in NPT were filled with previous values, because missing values can occur when the subject’s disease state is stable and clinicians find no need to perform redundant tests. To align the time points between MRI imaging and NPT, we selected the NPT measurements conducted closest to the date of each imaging visit. The time differences were on average 18.3 days, 462,3 days, and 433.1 days for AD, MCI, and CN subjects, respectively.

### Longitudinal change of brain volume and NPT scores

As our objective is to investigate AD progression, we focused on longitudinal changes in the volume of each brain region and in NPT scores. That is, we computed the volume changes (Δ brain volume) of 111 brain regions as the brain volume at the second visit minus the brain volume at the first visit (Fig. 1). Similarly, we computed change in NPT scores (Δ NPT scores) as NPT scores at the second visit minus the NPT scores at the first visit. We discarded increased values of brain regions (except ventricles) and decreased values of NPT scores. It is known that brain regions only shrink, and the increased volume may be due to the technical limitation of MRI acquisition that cannot distinguish CSF that fills the empty space after shrinking of neighboring brain regions. Some improved cognitive functions might be due to the effect of symptom medications, which cannot be seen as overall progression. In all, the inputs to the ph enotyping analysis were Δ i) a matrix *M* for brain volume loss with a shape of 857 subjects $\times \,$ 111 regions and (ii) a matrix *X* for NPT scores with a shape of 857 subjects $\times \,$50 tasks.

Because the volume for each brain region and scores in NPT tasks were on different scales, we normalized them to have values from 0 to 1 in by applying the normalizing function $f(x)=(x-min)/(max-min)$ in column-wise manner for all subjects. We did not use the original values of brain volume and NPT measures because we would like to solely focus on the amount of *change* rather than the original values that are already powerful indicator for the disease stages compared to the amount of changes.

### Computational phenotyping methods

Dimensionality reduction is one of the most widely used phenotyping methods; it can handle sparse and noisy data in heterogeneous healthcare data. Dimensionality reduction represents phenotypes as latent m edical concepts^46^. This means that phenotypes are defined as a probabilistic membership to medical components, and patients also have a probabilistic membership to the phenotypes. Nonnegative tensor factorization (NTF) is particularly popular due to its ability to model interactions between multiple data sources, its flexibility to adapt regularization methods, and the interpretability of latent medical concepts from its outputs^13–15^. The input for NTF is the interactions between different modalities (e.g., co-occurrence of medication and diagnosis within a time window). However, such explicit interactions sometimes are not observable. For this study, the interaction between volume changes in various brain regions and changes in cognitive functioning is the target output (rather than an input).

To overcome the lack of explicit interaction, we proposed a novel method based on coupled nonnegative matrix factorization (C-NMF)^47^. Our method is designed to cluster associated entities from either brain regions or cognitive tasks simultaneously so that the phenotypes can reflect both sides of information (i.e., both data modalities). Therefore, this method can capture interactions between the different modalities of data without explicit co-occurrence data, while retaining the advantages of NTF (i.e., interpretability and flexibility). Nonnegative matrix factorization (NMF) is a dimensionality reduction approach that represents the observed matrix $M$ as a low-rank latent dimension, which is interpreted in this study as the disease phenotypes. In NMF, *M* is decomposed into the product of two matrices $V$ and $Y$ that best approximate the original matrix. For this study, *M* contains the observed volume loss for the segmented brain regions, with a shape of (# patients) $\times$ (# brain regions); *V* contains the induced latent re presentation or membership of patients to phenotypes, with a shape of (# patients) $\times$ (# phenotypes); and *Y* represents the induced membership values of brain regions to each phenotype, with a shape of (# phenotypes) $\times$ (# brain regions). The objective function is then

$$L(V,Y)\,=\,||M-\,VY|{|}^{2}+\omega \cdot (||V|{|}_{1}+||Y|\,{|}_{1})$$

with nonnegative constraints ($V\ge 0$, $Y\ge 0$), ${l}_{1}\,$norm to shrink less important values and its weighting constant $\omega .$ Similarly, $X$ is decomposed into *V* and *W* where *X* contains the observed increased severity on each cognitive task, with a shape of (# patients) $\times$ (# tasks); *W* contains the membership values of cognitive tasks to each phenotype, with a shape of (# phenotypes) $\times$ (# tasks). The coupled nonnegative matrix fact orization (C-NMF) jointly factorize s two different observed matrices $M$ and $X$ assuming that they share the same dimension on patients and thus the same *V*:

$${L}_{NMF}(V,Y,W)\,=\,||M-VY|{|}^{2}+\,||X-VW|{|}^{2}+\omega \cdot (||V|{|}_{1}+||Y|{|}_{1}+||W|{|}_{1})$$

where $X$ is decomposed into *V* and *W*. Here, *X* contains the observed increased severity on each cognitive task, with a shape of (# patients) $\times$ (# tasks); *W* contains the membership values of cognitive tasks to each phenotype, with a shape of (# phenotypes) $\times$ (# tasks). Note that this co-factorization approach has the advantage of respecting the different distributions of the two data modalities, whereas factorization of one matrix of pooled sets of modalities does not.

One important characteristic that phenotypes should have is the ability to discriminate between the types of diseases under consideration, such as AD vs MCI, MCI vs CN, and AD vs CN. For this purpose, we added supervised regularization to the objective function^15,48^. Supervised regularization encourages the phenotypes to be separated according to the diagnostic label (i.e., AD, MCI, CN). Let us say $labe{l}_{ADvsMCI}$ is a binary indicator vector for AD and MCI (i.e., AD = 1, MCI = −1). The matrix $V$ contains patients’ membership values for the phenotypes, and ${V}_{ADvsMCI}$ is a subset of patients with either AD or MCI, to which the supervised regularization was applied. Note that we only used 80% of either AD or MCI patients to train the supervised regularizer. With a linear model ${\theta }_{ADvsMCI}$, a hinge loss function for AD vs MCI is

$$loss({V}_{ADvsMCI},\,labe{l}_{ADvsMCI}|{\theta }_{ADvsMCI})=max\{0,\,1-labe{l}_{ADvsMCI}\cdot f([{V}_{ADvsMCI},\,demo])\}$$

where $f([{V}_{ADvsMCI},\,demo])=\,{\theta }_{ADvsMCI}\cdot [{V}_{ADvsMCI},\,demo]+b$, *demo* is demographic features (i.e., time between two neuroimaging sessions, sex, and age), and *b* is a bias term. Note that we added demographic features to make sure that the discriminability of phenotypes is significant even after controlling for those demographic features. The supervised model *f*(·) is a linear support vector machine (SVM) that finds a balanced separating plane that distinguishes two groups. Similarly, we also separated AD vs CN as well as MCI vs CN. We then incorporate these supervised regularizers into the objective function:

$${L}_{sup}(V,\theta )\,=los{s}_{ADvsMCI}+los{s}_{ADvsCN}+los{s}_{MCIvsCN}$$

By jointly optimizing three independent linear SVMs, we can derive phenotypic representations that are discriminative among all three groups.

In all, a phenotype consists of a set of relevant brain regions and cognitive tasks. Each brain region or cognitive task contributes to the phenotype membership to a varying extent; for each phenotype, there is a row in Y and W containing the extent to which each region and task contributes to that phenotype. Likewise, every patient has a level of membership to each phenotype; for a given patient, the amount of membership to each of the phenotypes is stored in a row of *V*. We solved the optimization problem to minimize the objective function using Pytorch 11.4 optimizer with settings of adaptive gradient descent, maximum 1000 iterations, and an initial learning rate of 0.05.

### Evaluation on phenotyping methods

We evaluated the proposed phenotyping method in terms of its discriminative power and phenotype compactness^19^. We measured discriminative ability by the area under the receiver operating characteristic curve (AUC) metric to classify AD or MCI with the remaining patients that were not used to train the supervised regularizer. We computed separate AUC values to classify AD vs MCI, MCI vs CN, AD vs CN, and AD+MCI vs CN. Note that AUCs are used here to check how well the supervised regularizers work; the main point is to show that a multi-objective (classification & factorization) model like ours also has strong discriminative power. We measured compactness by sparsity and the degree of overlap between phenotypes. High sparsity means a few features (either brain regions or cognitive tasks) dominantly characterize phenotype whereas the other features are negligible, making clinical interpretation easier. The sparsity was computed as an averaged Gini index of involvement values in each phenotype (i.e., the rows of *Y* and *W*)^43^. The overlap measures the degree of overlapping between all pairs of phenotypes^19^. Phenotypes with less overlap are more distinctive for downstream clinical studies and interventions. The overlap is computed as an average of the cosine similarities between all pairs of column vectors of *Y* and *W*. We also computed the mean squared error (MSE) to evaluate how closely the derived phenotypes reflect the observed original data. We computed the mean and standard deviation from ten repeated trials (i.e., random resampling of train and test subjects). We compared the discriminative power and compactness of our proposed method with that of different settings of regularizers:

- C-NMF: Basic coupled NMF model without any regulariziers
- C-NMF + SVM: C-NMF with supervised regularizer based on linear SVM
- C-NMF +${l}_{1}\,$norm: C-NMF with ${l}_{1}\,$norm
- C-NMF +${l}_{1}\,$norm + SVM: C-NMF with ${l}_{1}\,$norm and SVM

We alternatively optimize NMF and SVM. We optimized the SVM every 200 iterations to balance the weight between NMF and SVM. The number of phenotypes *R* and the weight on ${l}_{1}$ norm regularizer $\mu$ were selected as 30 and 0.1 after many trials on the training/validation cohort; results are reported on the test cohort.

As a result, we found that our proposed regularized C-NMF outperforms other baselines in terms of discrim inative power and compactness. The C-NMF +${l}_{1}\,$norm and C-NMF +${l}_{1}\,$norm+SVM shows the highest AUCs, sparsity, and lowest overlap (Table 4). Particularly, the ${l}_{1}\,$norm regularizer significantly increased the discriminative power as well as the compactness.

**Table 4:** Comparison of discriminative power and compactness for various regularizing methods.

Met hods	Discriminability				Interpretability		Mean squared error	
	AD vs MCI	MCI vs.CN	AD vs. CN	AD+MCI vs CN	Sparsity	Overlap	Brain *M*	NPT tasks *X*
C-NMF	0.8108 (0.029)	0.7557 (0.0303)	0.9055 (0.0247)	0.8045 (0.0259)	0.7459 (0.0059)	0.2441 (0.008)	0.0028 (0.0001)	0.0 (0.0)
C-NMF + SV M	0.8059 (0.0335)	0.7404 (0.0601)	0.9056 (0.0224)	0.7938 (0.0443)	0.7507 (0.0089)	0.2395 (0.0099)	0.0029 (0.0001)	0.0 (0.0)
C-NMF + ${l}_{1}\,$norm	0.8035 (0.0338)	0.7981 (0.0493)	0.9001 (0.0283)	0.8287 (0.0406)	0.9189 (0.0066)	0.0611 (0.0065)	0.0055 (0.0002)	0.0003 (0.0)
C-NMF + ${l}_{1}\,$norm +SVM	0.7951 (0.0453)	0.8008 (0.0693)	0.9142 (0.0306)	0.8368 (0.0635)	0.9191 (0.0058)	0.0631 (0.0075)	0.0056 (0.0002)	0.0003 (0.0001)

We computed the average and standard deviation after 10 random resamplings of the train/test cohort.

## Supplementary information

Supplementary Information.

Supplementary Information2.

## Data Availability

The ADNI database is publicly accessible from adni.loni.usc.edu upon request.
