# Supplementary material for: Multimodal Phenotyping of Alzheimer’s Disease with Longitudinal Magnetic Resonance Imaging and Cognitive Function Data
Source: Sci Rep. 2020 Mar 26;10:5527. doi: 10.1038/s41598-020-62263-w (PMC7099007; doi:10.1038/s41598-020-62263-w)
Supplement: Supplementary file 1 — Supplementary Information. [file 41598_2020_62263_MOESM1_ESM.docx]

**Multimodal Phenotyping of Alzheimer’s Disease with Longitudinal Magnetic Resonance Imaging and Cognitive Function Data**

Yejin Kim, Ph.D.^1*^, Xiaoqian Jiang, Ph.D.^1^, Luca Giancardo, Ph.D.^1,2^, Danilo Pena, B.S.^1^, Avram S. Bukhbinder, B.S. ^2^, Albert Y. Amran, B.S. ^2^ and Paul E. Schulz, M.D.^2^;

for the Alzheimer’s Disease Neuroimaging Initiative^+^

^1^School of Biomedical Informatics, University of Texas Health Science Center at Houston, Houston, Texas, USA;

^2^Department of Neurology, the McGovern Medical School, University of Texas Health Science Center at Houston, Houston, Texas, USA;


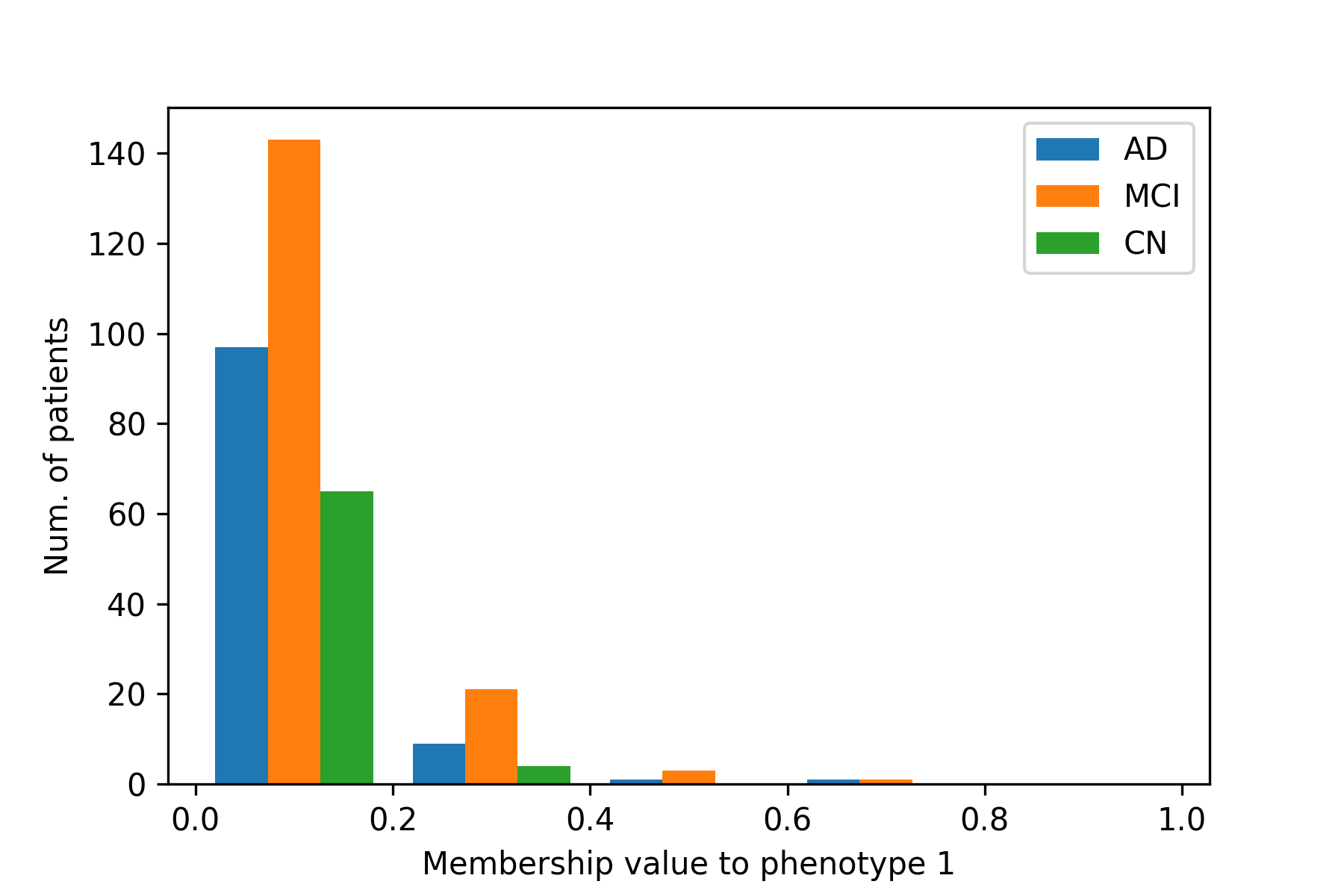

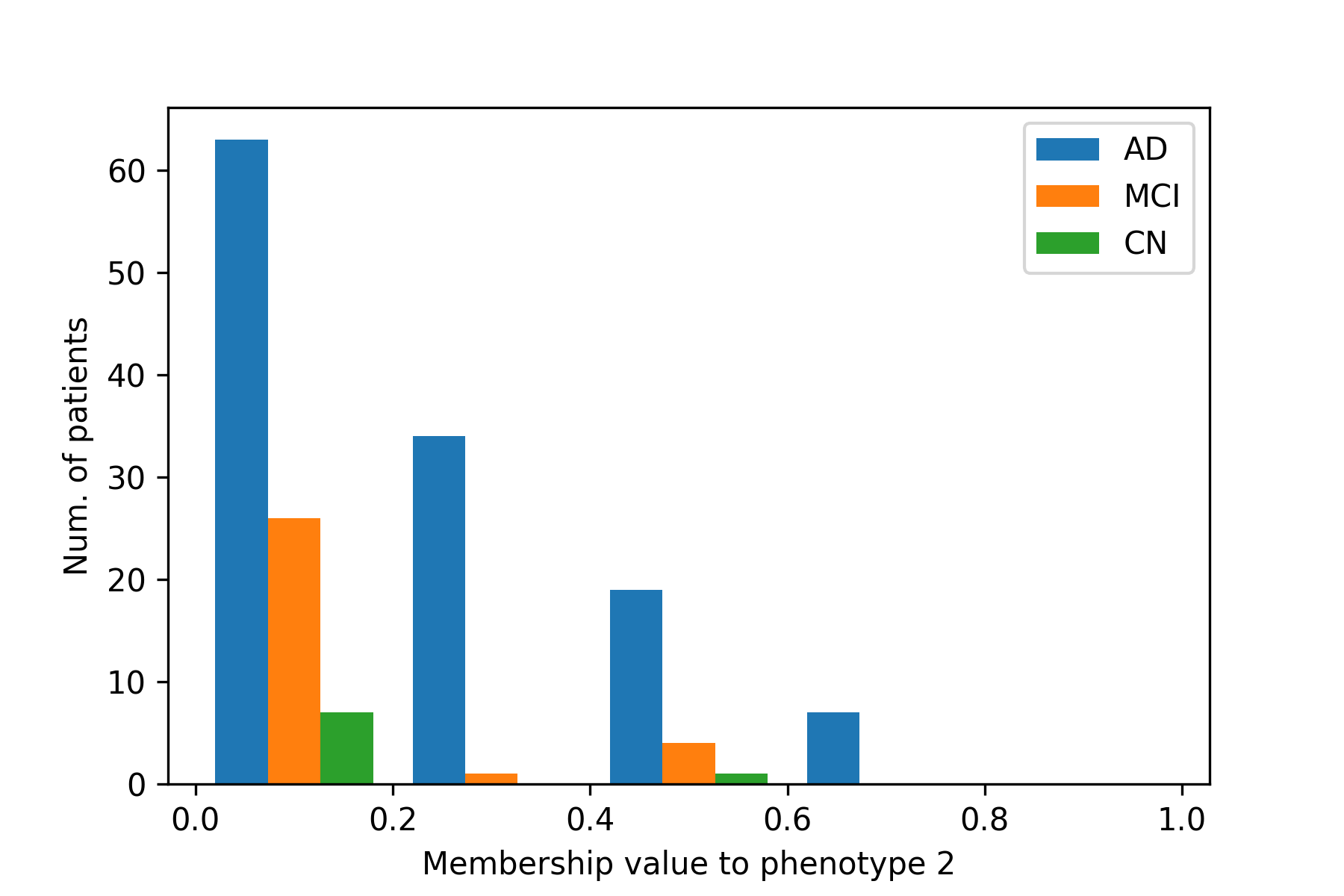

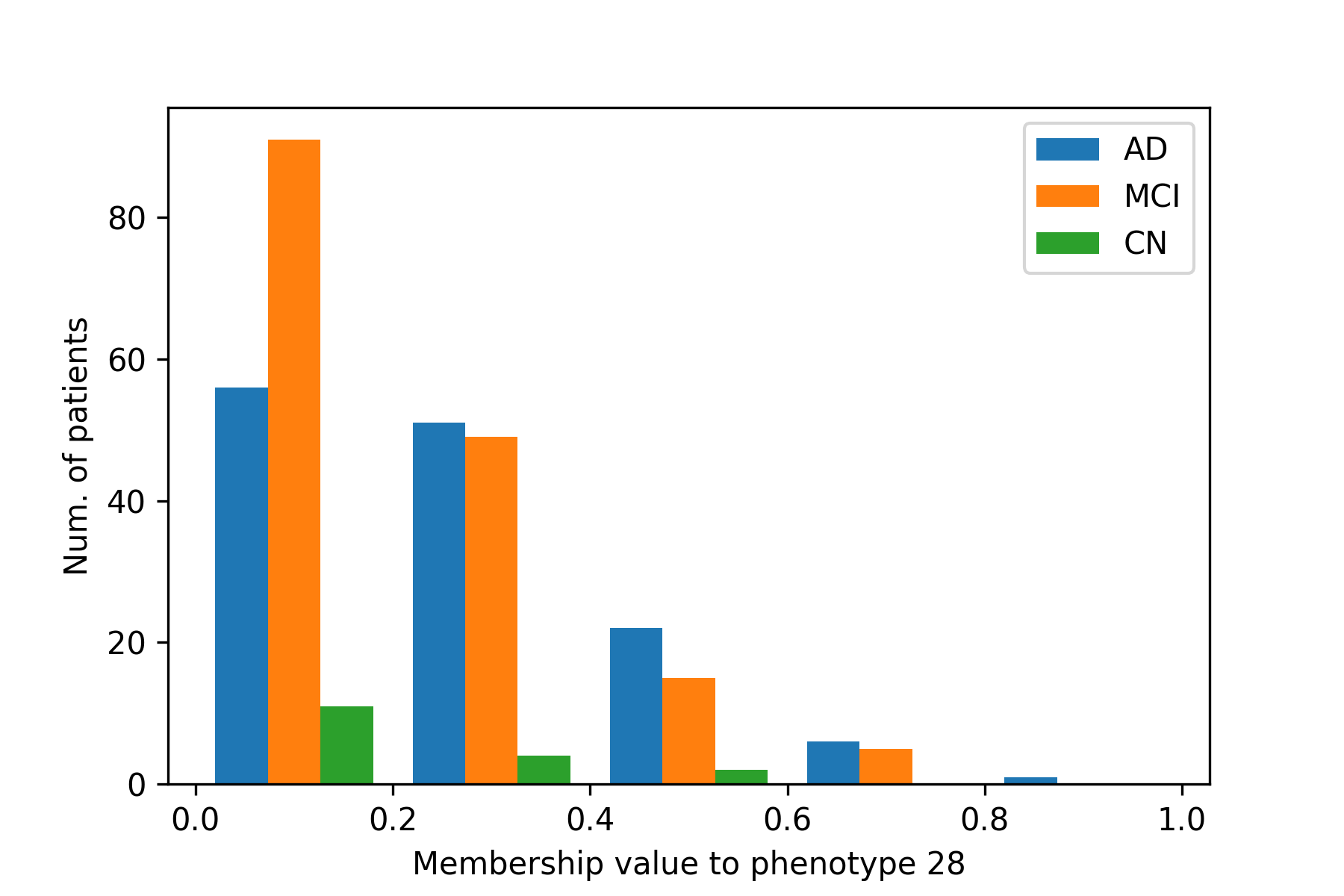

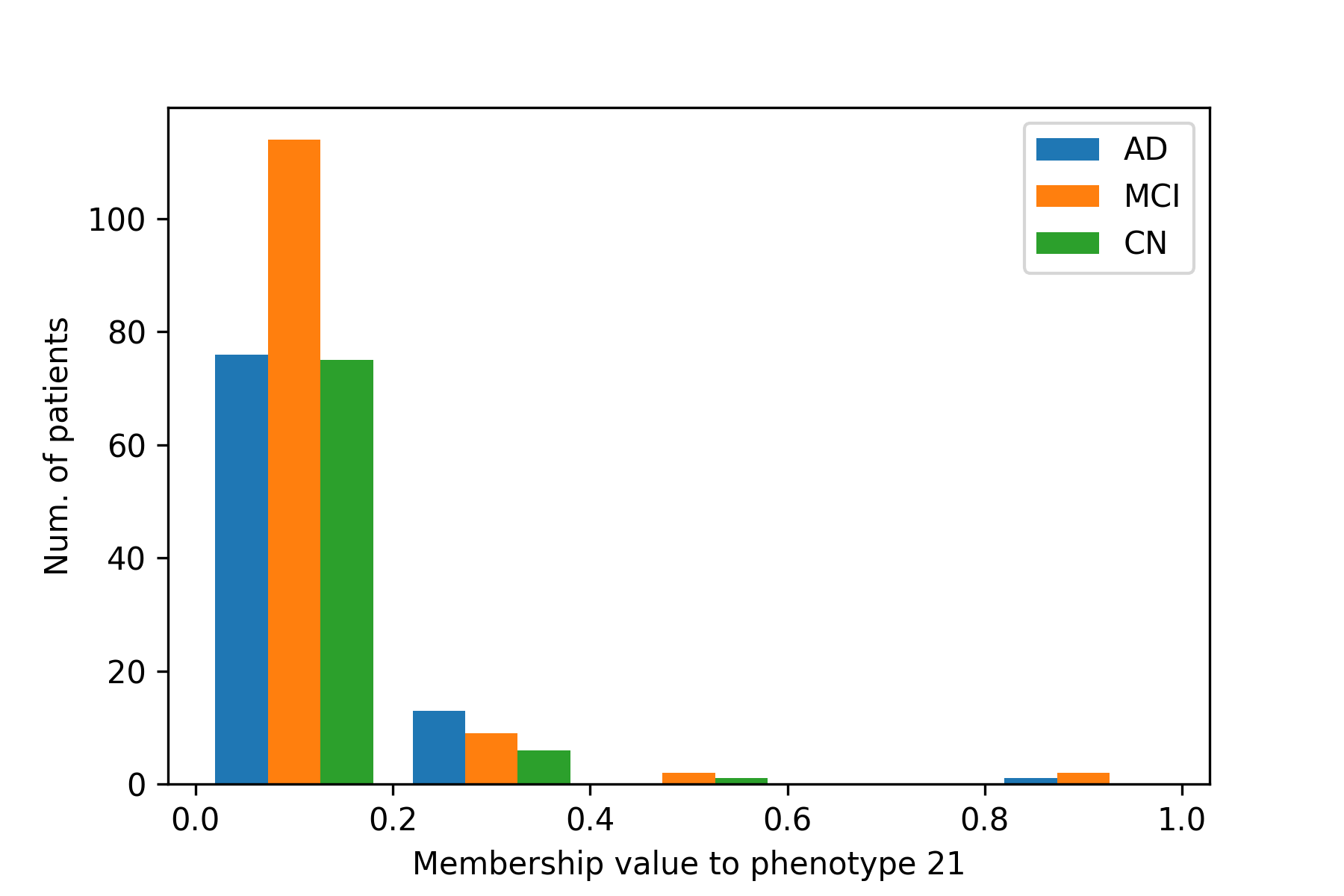

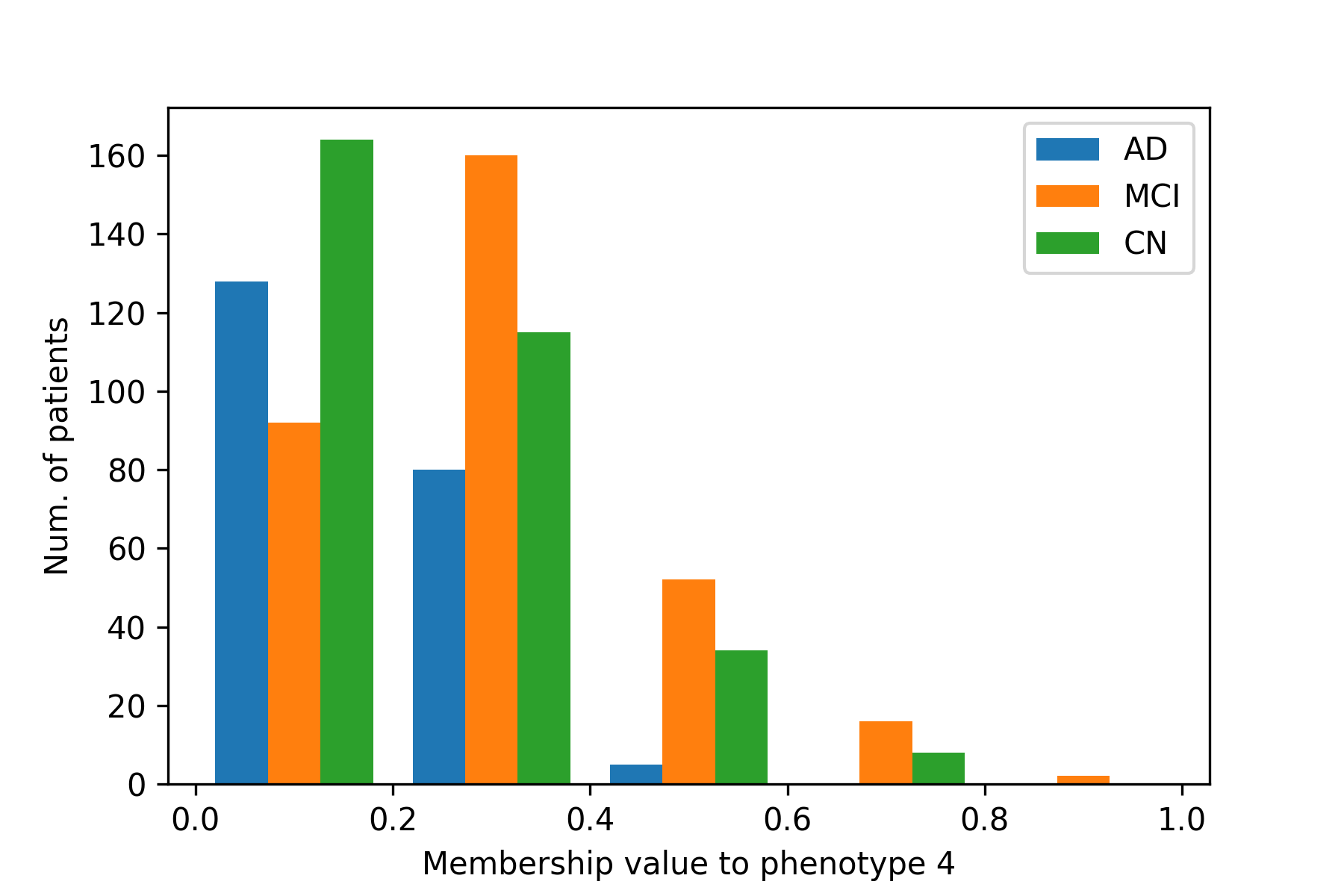


**Figure S1**. Distribution of AD, MCI, and CN subjects to the phenotypes. The proportion of AD patients to MCI or CN patients increases as the membership value increases in Phenotype 2 (P2). The proportion of patients with AD or MCI to patients who are CN increases as the membership value increases in P28, P1 and P21.

**Table S1.** Number of transitions from one stage to another.

|  | | Visit 2 | | |
| --- | --- | --- | --- | --- |
|  |  | AD | MCI | CN |
| Visit1 | AD | 209 |  |  |
|  | MCI | 159 | 148 | 14 |
|  | CN | 16 | 34 | 263 |

**Table S2.** Discriminability of phenotypes. We examined discriminability of phenotypes using logistic regression coefficient and *p*-value on classifying AD and/or MCI versus CN. Prevalence = (the number of patients whose membership to the phenotype is greater than $10^{-5}$ [i.e., $>0$] divided by the total sample size [i.e., 857]) $\times$ 100. The coefficient and p-value for classifying AD vs CN were not listed because AD and CN were completely separated. We selected AD phenotypes if (coefficient of AD vs MCI classifier > 0 AND p-value <0.05) AND (coefficient of AD+MCI vs CN classifier >0 AND p-value <0.05); MCI phenotypes if (coefficient of AD vs MCI classifier <0 AND p-value<0.05)AND(coefficient of MCI vs CN classifier >0 AND p-value <0.05); CN phenotype if (coefficient of MCI vs CN classifier <0 AND p-value<0.05)AND(coefficient of AD+MCI vs CN classifier <0 AND p-value<0.05).

| Phenotypes | AD vs MCI | | MCI vs CN | | AD+MCI vs CN | | Prevalence (%) |
| --- | --- | --- | --- | --- | --- | --- | --- |
|  | Coef. | p-value | Coef. | p-value | Coef. | p-value |  |
| P2 | 8.12 | 0.00 | 14.42 | 0.02 | 15.11 | 0.01 | 18.90 |
| P28 | 4.67 | 0.00 | 4.85 | 0.05 | 5.03 | 0.04 | 36.76 |
| P1 | 0.98 | 0.67 | 10.35 | 0.04 | 10.24 | 0.04 | 40.26 |
| P20 | -1.16 | 0.50 | 16.40 | 0.00 | 16.61 | 0.00 | 32.09 |
| P21 | -0.70 | 0.75 | 10.30 | 0.02 | 10.32 | 0.02 | 34.89 |
| P6 | -9.86 | 0.05 | -5.13 | 0.20 | -5.00 | 0.21 | 14.70 |
| P12 | 0.59 | 0.54 | 5.79 | 0.00 | 5.68 | 0.00 | 43.64 |

**Table S3 Full phenotype list**

(Please refer to separate Excel file)
